# Both Lexical and Non-Lexical Characters Are Processed during Saccadic Eye Movements

**DOI:** 10.1371/journal.pone.0046383

**Published:** 2012-09-28

**Authors:** Hao Zhang, Hong-Mei Yan, Keith M. Kendrick, Chao-Yi Li

**Affiliations:** 1 Key Laboratory for NeuroInformation of Ministry of Education, University of Electronic Science and Technology of China, Chengdu, China; 2 Shanghai Institutes of Biological Sciences, Chinese Academy of Sciences, Shanghai, China; University of Muenster, Germany

## Abstract

On average our eyes make 3–5 saccadic movements per second when we read, although their neural mechanism is still unclear. It is generally thought that saccades help redirect the retinal fovea to specific characters and words but that actual discrimination of information only occurs during periods of fixation. Indeed, it has been proposed that there is active and selective suppression of information processing during saccades to avoid experience of blurring due to the high-speed movement. Here, using a paradigm where a string of either lexical (Chinese) or non-lexical (alphabetic) characters are triggered by saccadic eye movements, we show that subjects can discriminate both while making saccadic eye movement. Moreover, discrimination accuracy is significantly better for characters scanned during the saccadic movement to a fixation point than those not scanned beyond it. Our results showed that character information can be processed during the saccade, therefore saccades during reading not only function to redirect the fovea to fixate the next character or word but allow pre-processing of information from the ones adjacent to the fixation locations to help target the next most salient one. In this way saccades can not only promote continuity in reading words but also actively facilitate reading comprehension.

## Introduction

While reading this paper, you are continually making ballistic, saccadic eye movements, which serve to bring a new region of text into foveal vision for detailed analysis. Every saccade is associated with a transient but high speed displacement of the retinal image. A large number of studies have shown that visual stimuli presented just before and during saccades are not perceived [1], [2], [3]. This reduced visual performance is attributed to so called saccadic suppression or saccadic omission, which results in us being unaware of any blurring of viewed images during perception and maintaining visual stability. However, saccadic suppression is proved to be selective, for instance, stimuli of low spatial frequencies are very difficult to detect if flashed just prior to a saccade, while stimuli of high spatial frequency and equiluminance remain equally visible or even more sensitive [4], [5]. There may also be saccadic compression of both spatial and temporal aspects of visual processing [6], [7], [8]. The neural mechanism of saccadic suppression and compression is still not very clear.

A key additional question is whether higher level cognitive processing might also be suppressed during saccades. To date researches have shown that saccades disrupt counting [9], judgments of numerical magnitude [10], [11] and spatial direction [12], mental rotation [13], and some shifts of spatial attention [14] but not other cognitive processes, such as the integration of visual-form information [15] and perceptual stability [16].

In the field of cognitive linguistics there is an ongoing and long-standing debate about whether lexical processing during reading is also suppressed during saccades. Some studies such as EZ reader model have concluded that information processing only occurs during period of eye fixation and not during the execution of saccades [17]. This is in line with claims that during saccades the eyes are moving so quickly across characters and words that only a blur would be perceived [18], [19]. This view has led to saccade duration conventionally being subtracted from reading comprehension time in most eye movement experiments. However, other studies investigating sentence reading and text comprehension have suggested that higher-level cognitive processing such as lexical processing might not be completely be suppressed during saccades [20], [21], [22]. Overall therefore we still do not have a clear idea whether linguistic information is being actively processed during saccades [23], [24] and this is clearly an important and fundamental question in this field which needs to be definitively resolved.

We have therefore investigated whether both words (lexical - Chinese) and no intrinsic meaning (non-lexical - alphabetic) characters could be processed or not during fast saccadic eye movement. Our results provide the first evidence that discrimination of both these lexical and non-lexical characters is remarkably accurate for those scanned during the saccade towards the next fixation point, but is considerably less so for those not scanned by the saccade beyond the fixation point. Discrimination performance for the characters scanned during the saccade is almost similar to that during actual fixation, although with the latter discrimination of characters after the fixation point is considerably better. The functional significance of these results in guiding the next fixation position and in facilitating information processing is also discussed.

## Materials and Methods

### Subjects

Six subjects (5 male, 1 female) aged 21–24 yr participated in the experiments. All subjects were native Chinese speakers although they all had some knowledge of spoken and written English. All subjects had normal or corrected-to normal vision and provided written informed consent. All research experiments were approved by the Ethics and Human Participants in Research Committee, University of Electronic Sciences and Technology of China, Chengdu, China. One of the subjects was an author of this study while the others were naïve with respect to its aim. All the subjects were given an initial training period of about 30 min before the experiments in order to be familiar with the task. Data from this training session were not included in the final analysis. Each subject performed in two sessions for each experiment, and each session included 10 blocks resulting in a total of 840 individual trials.

### Experimental setup

The subjects were seated in a dark room specially designed for psychophysical experiments. The visual stimuli were presented on a 21″ color monitor (DELL Trinitron) providing a frame frequency of 100 Hz at a spatial resolution of 1280×1024 pixels. The viewing distance was 80 cm, and stimuli appeared on a grey background which was adjusted to a mean luminance of about 22 cd/m^2^. Originally the contrast of both Chinese and Alphabetic characters were 1.0 however preliminary tests showed that at this level discrimination performance on Alphabetic characters showed a ceiling effect which would prevent comparisons across viewing conditions and so task difficulty was increased by decreasing the contrast to 0.5.

Eye movements were recorded with an infrared eye tracker (Eyelink2000, SR Research Ltd.) and sampled at 1000 Hz. Head movements were restricted by a forehead and chin rest. The pupil of the left eye was tracked at a sample rate of 1000 Hz and a spatial resolution of 0.1°.

### Stimuli

Two sets of stimuli were used in the experiments. The first set consisted of 30 Chinese characters which were all composed of 4 strokes and had the same usage frequency [25]. The second set comprised 25 capital alphabetic characters (Q was excluded to avoid confusion with O). All the characters were displayed on the screen at the same size (0.5°×0.5°). For each trial, fourteen characters (with no lexical or semantic relationship) were randomly chosen with seven on each side of the scheduled fixation point. The one which was chosen as the target for discrimination was displayed in bold face. The distance between each character was 0.2°.

### Experimental paradigm

**Figure 1:**
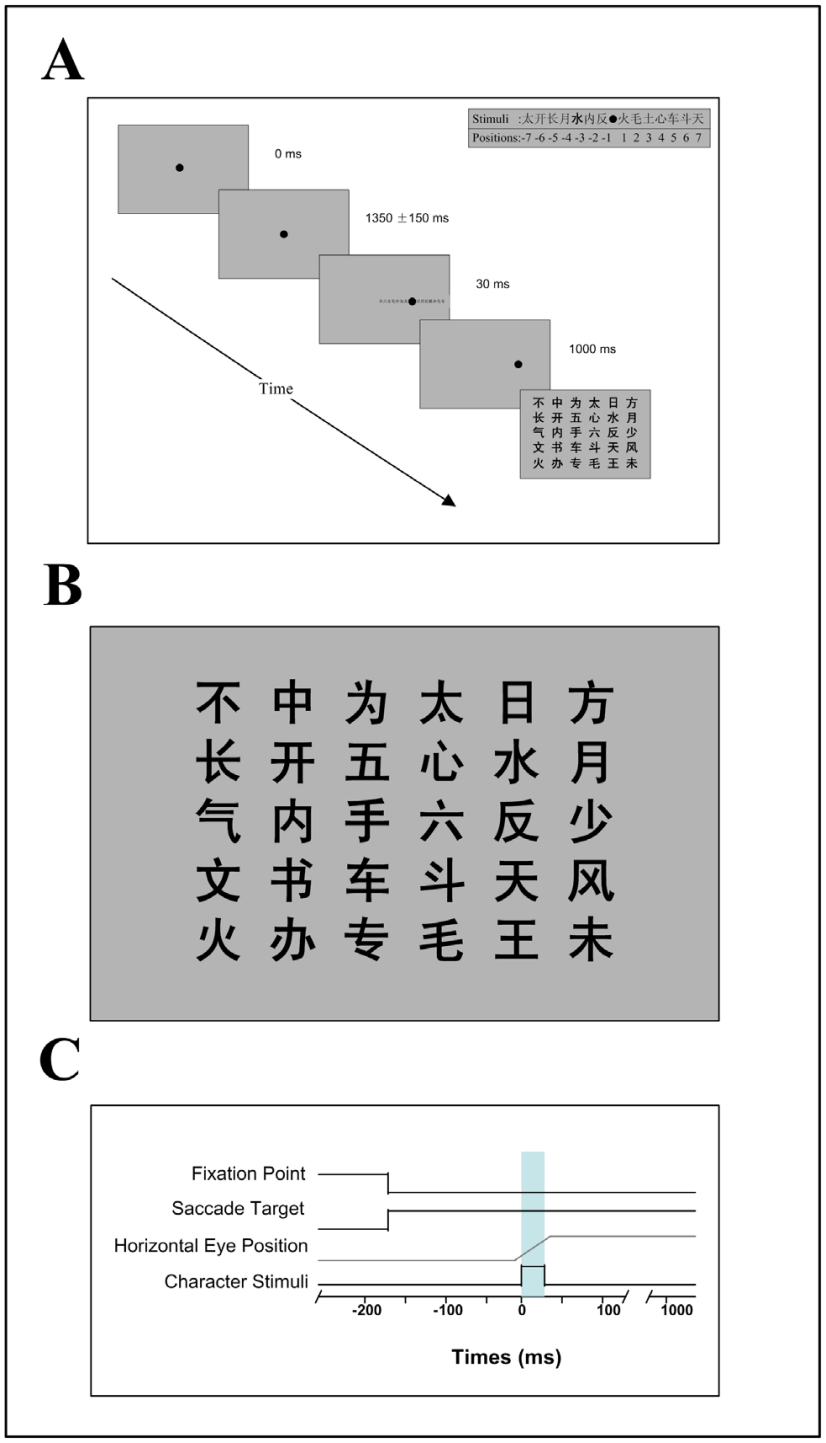
Paradigm for the saccadic eye movement experiment showing the stimulus sequence (A) and timing (C). An example of a Chinese character stimulus set is shown at the top right of A, and the blue bar in C marks presentation duration of the character stimulus. (B) shows all 30 Chinese stimulus characters used in the study.

The main experiment (Experiment 1) consisted of two recognition tasks involving: (a) Chinese character discrimination and (b) Alphabetic character discrimination. The two tasks had the same experimental protocol. Fig. 1A shows the procedure for the Chinese character discrimination task. Both the fixation dot and stimulus characters were presented on a CRT screen against a homogenous gray background. Each trial started with the appearance of a black fixation dot (0.3° in diameter) at the center of the screen, which was extinguished after 1200∼1500 ms and followed by the appearance of a dot occurring randomly at either 7° on the left or 7° on the right of the center fixation point, indicating the goal of the saccade (0.3° in diameter). At this point, subjects were required to perform a saccade from the fixation point (FP) to the goal point (saccadic target, ST). The direction of the saccadic goal point was varied randomly within each block of trials. Eye positions were recorded and the velocity of eye movement was calculated on line.

We used the initiation of eye movement to trigger the presentation of character stimuli. As soon as the stimulation program detected the initiation of a saccade (velocity≥35°/s) [26], fourteen characters were presented for 30 ms, seven on each side of the goal point. As timing is critical in the experiment, we determined the delay from eye movement to stimulus update to be about 10 ms in our system. The average duration of saccade execution is about 40∼50 ms, during which the character stimuli were presented and the recognition task performed. About 1000 ms after the saccade, a panel including all the test characters was shown on the screen (Fig. 1B), and the subjects were required to pick out the appointed character using a computer mouse. When the subjects had made their choice, the next trial started. The temporal sequences for the experimental protocol are shown in Fig. 1C.

In order to establish discrimination performance relative to fixation and immediately following saccadic eye movement, two further experiments were carried out. In Experiment 2, Chinese and Alphabetic character discrimination was investigated during steady fixation. All stimuli were presented on a homogeneous grey background. A fixation dot (0.3° in diameter) was presented at the center of the display screen and subjects were instructed to maintain fixation on this point during the trial. After 1200∼1500 ms, 14 Chinese or alphabetic characters were presented on either side (7 each side) of the fixation point for 30 ms. The character panel was then shown on the screen 1000 ms later and subjects were required to pick out the appointed one. Experiment 3 was similar to that for character discrimination during saccadic execution, but the characters were not presented until 100 ms after saccade initiation, namely, when the eyes steadily re-fixated the saccadic landing position.

To better understand the function of active saccades in reading, a control experiment was carried out where the eye was exposed to a moving word without actually making a saccade (i.e. the equivalent of a passive saccade)(Experiment 4). The procedure was similar to that for character discrimination during the steady fixation condition. During the trials, when a fixation dot was presented at the center of the display screen, subjects were instructed to maintain their fixation on this point. After 800∼1100 ms, fourteen characters with normal type face were presented either 7.5° on the left or right of the center fixation point and moved towards the direction of the fixation point at a speed of 30°/s. This low speed was deliberately chosen so it would make potential information processing easier than with higher ones. After the characters had moved for 230 ms, one of them was displayed in bold face for 30 ms and swept across the fixation point. The characters were presented in normal type face again and continued to move until the end point of 7.5° opposite to the starting position. About 1000 ms later, the subjects were required to pick out the appointed character using the computer mouse. In this way we could determine whether visual information processing during reading requires the making of an active saccadic movement or can also occur when character information passes across the retina for the same amount of time and speed as for very slow saccade.

### Data analysis

To trigger the presentation of character stimuli, eye movement velocity was calculated on line for every trial. Saccade initiation time was generally determined by a velocity criterion with a threshold of 10% of the maximum speed during the saccade (≥35°/s in this case). Trials were discarded when any one of the following situations occurred: (a) no saccade; (b) multiple saccades; (c) a blink during the trial; (d) unsteady saccadic landing positions in which the eye positions from 100 ms to 300 ms after saccadic initiation fell outside 1° from the saccade target point.

### Statistics

In all the 4 experiments Chi-square was used to calculate the level of discrimination performance that was significantly better than chance. With 60 discrimination trials for each character position and 30 possible Chinese characters and 25 possible alphabetic ones it was calculated that >20% choice accuracy was required for p<0.05. Comparisons between discrimination performance within the first three experiments were made using a 3-way repeated measure ANOVAs (factors: character position, before/after the fixation point and saccade direction). For the fixation condition a 2-way ANOVA was performed since there was no saccade direction factor. Comparisons across experiments were also made using a 3-way ANOVA with experiment type, character position and before/after fixation point as factors. No comparisons were made between performance on Chinese and Alphabetic character sets since the character contrasts were intentionally different. ANOVA and post-hoc multiple comparison tests (LSD and Student- Neuman-Keuls) were carried out using SPSS software.

## Results

### Discrimination of Chinese and alphabetic characters during saccadic eye movement

**Figure 2:**
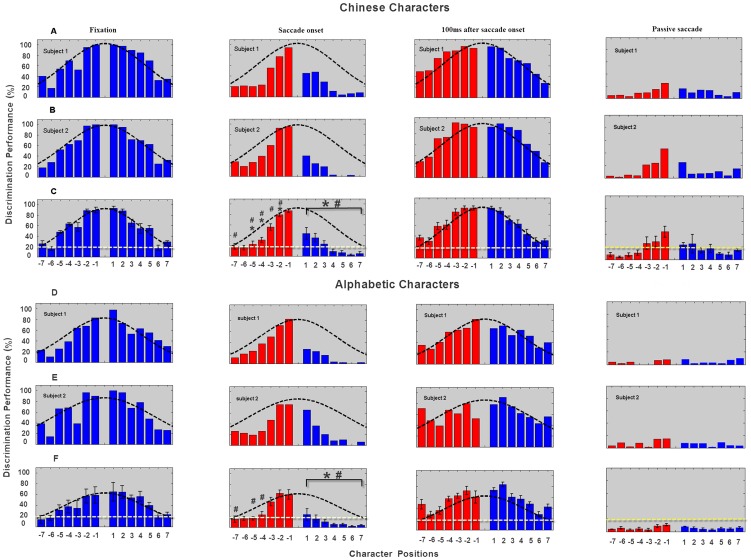
Discrimination performances of Chinese and Alphabetic characters during the four viewing conditions. First column, data were obtained during steady fixation. Second column, data were obtained during saccadic execution. Third column, data were obtained 100 ms after saccadic onset. Last column, data were obtained from control experiments with passive saccade moving at a speed of 30°/s. For each condition, A, B and D, E show data from two representative subjects and C, F, the mean of all the six subjects, the bars indicate ± sem. Numerals on the x-axis show the positions of the characters relative to the fixation point (0). In each case red bars show characters tracked by a saccade and the blue bars characters not tracked by a saccade. The black dashed lines showed the fitted Gaussian curves and the white dashed line in C and F indicates the threshold for significantly better than chance performance (Chi-square). * p<0.05 compared with the same character position during fixation and # p<0.05 during the 100 ms post saccade condition.

The discrimination performances for Chinese characters during saccadic movement execution are shown in Fig. 2A–C (Second column) and were significantly above chance for the 5 characters nearest the fixation point scanned by the saccade and the 3 characters after the fixation point not scanned by the saccade. A 3-way ANOVA showed that subjects' discrimination of Chinese characters varied significantly for both character position (F_6,30_ = 56.6, p<0.0001) and side of the fixation point (F_1,5_ = 33.7, p = 0.002), with characters scanned by the saccade before the fixation point being discriminated better than those not scanned by the saccade after it. Post-hoc analysis showed that the first 3 characters scanned by the saccade before the fixation point were discriminated significantly better than those in the other 4 positions (p<0.0001 in each case). There was a trend towards significance for saccade direction to influence discrimination performance (F_1,5_ = 5.977, p = 0.058), but no significant interaction with either character position (F_6,30_ = 1.778, p = 0.138) or side of the fixation point (F_1,5_ = 1.364, p = 0.296) was found. For alphabetic characters discrimination performances during saccadic movement execution are shown in Fig. 2D–F (Second column). The first 4 characters before and only the first after the fixation point were discriminated better than chance. There was a similar significant effect of character position (F_6,30_ = 19.75, p<0.001) and side of the fixation point (F_1,5_ = 59.31, p = 0.001) with again the first 3 characters before the fixation point being discriminated better than the other 4 (p<0.001). Here there was clearly no significant effect of saccade direction (F_1,5_ = 0.137, p = 0.727). Therefore in Fig. 2, data of saccades from right to left and left to right were combined according to their corresponding positions on scanned or unscanned side respectively.

**Figure 3:**
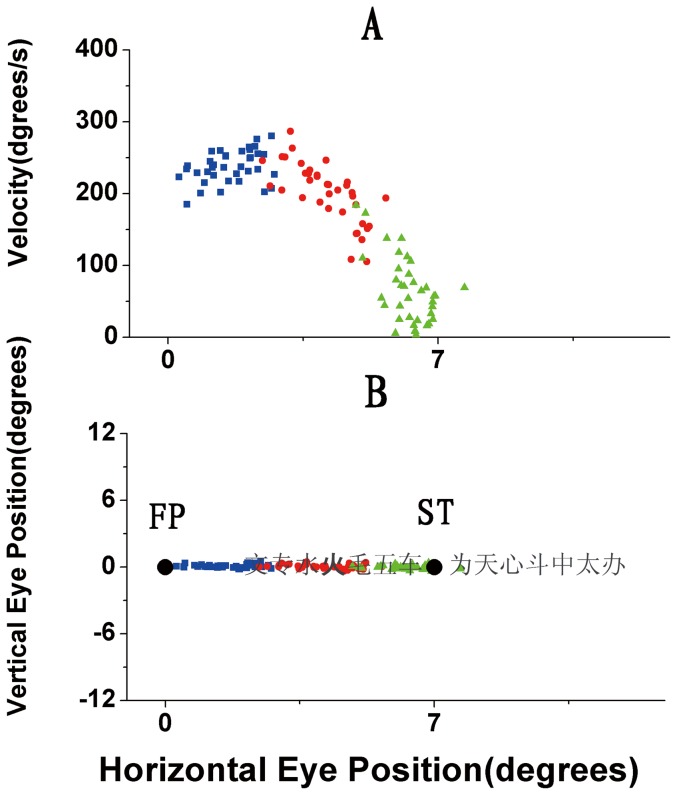
Velocities of the saccadic movements (A) and the corresponding positions of the eyes across the sample stimuli (B) during the presentations of character stimuli for all trials in a block. The screen frame frequency was 100 Hz and so during the 30 ms presentation of character stimuli there were three frames, and the eye movement velocity and position were calculated for each frame. The sequence of the three frames is marked with different colors (blue, red and green) and the dots represent transient velocity and position of the eyes from the first, second and third frame, respectively.

Since the presentation of character stimuli was triggered by saccade initiation and the average duration of saccade execution was about 40∼50 ms, during the period of character presentation (30 ms) the eyes were moving at high speed. Fig. 3 illustrates one subject's the actual velocities of the saccadic movements (A) and the corresponding positions of the eyes across the sample stimuli (B) during the presentations of character stimuli for all trials in a block. Other subjects' data are similar with Fig. 3. From Fig. 3 it can be seen that eye movements reached a peak velocity of around 250°/s during the first stimulus frame (blue dots) and decreased to around 200°/s during the second frame, then the eye velocity fell to about 80°/s during the third frame. Our experiments therefore showed that the visual system was capable of very good character discrimination performance even when the eyes were scanning at rather high speeds during saccades.

### Discrimination of Chinese and alphabetic characters during steady fixation

Fig. 2 (First column) shows that discrimination accuracy for the Chinese (A–C) and alphabetic (D–F) characters was significantly above chance for the 5 characters on either side of the fixation point with performance decreasing with distance from it. For Chinese characters a 2-way ANOVA showed that discrimination performance varied significantly across character positions (F_6,30_ = 169.28, p = <0.001) but not for side of the fixation point (F_1,5_ = 0.677, p = 0.442). For Alphabetic characters there was also a significant effect of character position (F_6,30_ = 18.89, p = 0.002), and there was also a significant effect of side (F_1,5_ = 14.432, p = 0.013) with characters to the right of the fixation point being discriminated better than those on the left. The fitted Gaussian curves implicated that the performance of both sides is symmetrical during steady fixation.

### Discrimination of Chinese and alphabetic characters after saccadic movement

Figs. 2 A–F (Third column) show that when Chinese and alphabetic characters were presented 100 ms after saccade initiation when the eyes steadily re-fixated the saccadic landing position, discrimination performance was above chance for all character positions either side of the fixation point. A 3-way ANOVA showed that there was a significant effect of character position (Chinese - F_6,30_ = 58.53, p<0.0001; alphabet - F_6,24_ = 18.33, p<0.0001) with characters in the first 2–4 positions being discriminated best. The ANOVA also showed a significant effect of side with characters before the fixation point scanned by the saccade being discriminated slightly better than those after it which were not scanned (Chinese - F_1,5_ = 13.26, p = 0.015; alphabetic - F_1,4_ = 8.96, p = 0.04). Thus accurate discrimination performance during saccades is still slightly biased towards characters actually scanned by the saccade.

### Comparison of discrimination of Chinese and alphabetic characters during saccades with post-saccade and fixation performance

Comparisons between discrimination performances in the three different viewing conditions were made using a 3-way ANOVA. This revealed that there were significant differences across the three conditions for both the Chinese (F_2,10_ = 31.281, p = 0.000) and Alphabet (F_2,8_ = 22.519, p = 0.003) character discrimination, with performance during the 30–40 ms saccade being significantly worse than for the fixation (p = 0.001 and p = 0.073) and 100 ms post-saccade (p = 0.005 and p = 0.001) conditions but not between the latter two (p = 0.746 and p = 0.295). There was also a significant overall difference for discrimination of the 7 characters either side of the fixation point for both Chinese (F_6,30_ = 170.787, p = 0.000) and Alphabetic (F_6,24_ = 42.872, p<0.001) sets. Here Chinese characters viewed during the 30–40 ms saccade track were slightly less well discriminated than in the fixation (p = 0.012) and 100 ms saccade (p = 0.04) conditions. The performances of characters after the fixation point which were not tracked by saccades were significantly lower than in the fixation (p = 0.002) and 100 ms post-saccade condition (p = 0.003). There were no differences between the fixation and 100 ms post saccade conditions (p = 0.308 and p = 1.000) for characters before and after the fixation point respectively. For the Alphabetic characters there was no significant overall difference between discrimination performance of fixation vs saccadic execution conditions for characters before the fixation point (p = 0.593) due to the easy task compared with Chinese character, while there was significant difference between saccadic and post-saccadic conditions (p = 0.012). For characters after the fixation point performance was significantly worse in the 30–40 ms saccade condition compared with the fixation (p = 0.003) and 100 ms post saccade conditions (p<0.0001). There were no differences between the fixation and 100 ms post saccade conditions (p = 0.086 and p = 0.159) for characters before and after the fixation point respectively.

### Discrimination of Chinese and alphabetic characters during passive saccades

The last column of Fig. 2 shows the results obtained during the experiment of passive saccades (see Materials and Methods). The discrimination accuracy for Chinese characters (the upper three plots of the right column) was around chance (20%) when the passive saccade was set at a speed of 30°/s. In this case, only the nearest character before the fixation point scanned by the passive saccade was discriminated slightly above chance. At the same speed, the performance for alphabetic character discrimination was even lower. The discrimination accuracy at all positions was lower than chance with a mean of less than 10% accuracy. 2-way ANOVA showed that the performances of tracked side during passive saccade were significantly less than those of saccadic condition for both Chinese and alphabetic characters (p = 0.015 for Chinese characters, p = 0.002 for alphabetic characters, respectively). The performances of Chinese characters on unscanned side were aslo significantly lower than those of saccadic condition (p = 0.024). For alphabetic characters on unscanned side, there was no significant difference between passive saccades and active saccades (p = 0.133), because both of them were close to the chance level.

## Discussion

Previous studies have reported that lexical processing may take place during saccades [20], [22]. Since all these experiments were conducted with meaningful sentences, the semantic relationship between words or sentences may also contribute to information processing or text comprehension and thus be confused with the effects of saccades. In the present experiments, the stimuli characters we presented have no lexical or semantic relationship between them, just character strings. Our results show the first clear evidence that information from both lexical and non-lexical characters is processed during saccadic eye movements even though the eye is moving at very high speed. Moreover, the fact that discrimination accuracy is significantly better for characters scanned than those not scanned indicates that the information processing is biased towards the path actually scanned. Furthermore, under conditions where subjects were exposed passively to character information mimicking that which would occur during the making of a very slow saccade, they could not discriminate lexical or non-lexical characters. Therefore, our experiments indicate that the information processing during saccades is based on some active mechanisms that may be related to space-directed attentional control. Our brain tends to pay more attention to important visual information near the next saccadic target and that on the scanned path. Thus remarkably, contrary to the previous assumption that at such high speeds information processing would be impeded due to blurring and require suppression, the human eye and brain are quite capable of overcoming this supposed obstacle. This opens up the possibility that saccades do not function simply to bring a fixation point to the center of the retina but can also aid information processing of characters scanned on the way towards it.

Some previous work has also suggested that visual information can be processed during saccades. Recently, Watson and Krekelberg [16] employed a visual shape illusion to probe the mechanism underlying saccades. They found that an inducing stimulus (an oriented line) presented unconsciously just before a saccade distorted the perceived shape of a subsequently presented circle. The implication of their findings is that the visual brain continues to process visual information during saccades but that observer is not aware of this. Our experiments however extend beyond these findings to provide direct evidence that lexical Chinese characters as well as simpler non-lexical alphabetic ones are actually accurately discriminated during saccades.

While it is possible that there is an element of covert processing of characters by the subjects during saccades in our study they all reported awareness of the characters tracked, especially those close to the fixation point. The high level of discriminatory performance achieved for the first 3 characters tracked by the saccade before the fixation point also argues strongly that an element of conscious processing was involved. Indeed, the sharp drop off in performance for characters more than 3 positions away from the fixation point is suggestive that these might be being processed more covertly. Interestingly, this also represented the main difference from the fixation and 100 ms post-saccade conditions where discrimination performance of characters 4 and 5 positions away from the fixation point was significantly better.

In the field of cognitive linguistics this opens up the fascinating possibility that during reading the visual system is in fact routinely processing character information near the saccadic target (re-fixation point) during the saccade execution. Thus information processed during saccades should influence word recognition and also serve potentially to aid comprehension and guide the choice of subsequent fixation points. This kind of information process may be caused by receptive fields shifting of neurons or attention remapping during saccades [27], [28], [29].

Our results showed that character discrimination performances beyond the fixation point recovered to the level seen during fixation by 100 ms after the initiation of a saccade. This is consistent with previous findings showing that visual performance of some aspects is recovered even enhanced 50–100 ms after saccade onset [4], [5], [30]. Electrophysiological recording experiment has also proposed that visual responsiveness is enhanced after saccades, and concluded that saccades not only provide a means to shift gaze direction but also to increase visual sensitivity when saccades are frequent [31]. When reading, for example, we do make a high frequency of saccadic eye movements. As Ibbotson and Cloherty [32] have suggested, rather than suppressing everything during a saccade, the visual system is geared towards enhancing what happens after it by facilitating the processing of information near the saccadic target (re-fixation point). This mechanism of information processing during saccades may provide a simple and elegant strategy to maintain visual stability and continuity while incorporating useful and predictive information from subsequent fixations.

In conclusion, our results suggest that saccades during reading not only function to redirect the fovea to fixate the next character or word but allow pre-processing of information from ones adjacent to the previous fixation to help target the next most salient one. In this way saccades may not only promote stability and continuity in reading words, but also actively facilitate reading comprehension.
